# Endogenous peripheral oxytocin measures can give insight into the dynamics of social relationships: a review

**DOI:** 10.3389/fnbeh.2014.00068

**Published:** 2014-03-11

**Authors:** Catherine Crockford, Tobias Deschner, Toni E. Ziegler, Roman M. Wittig

**Affiliations:** ^1^Department of Primatology, Max Planck Institute for Evolutionary AnthropologyLeipzig, Germany; ^2^Wisconsin National Primate Research Center, University of WisconsinMadison, WI, USA

**Keywords:** endogenous peripheral oxytocin, non-invasive sampling, multi-hormone sampling, social bonds, social interaction dynamics, cooperation

## Abstract

The neuropeptide, oxytocin, receives increasing attention due to its role in stress regulation and promoting affiliative social behavior. Research across mammals points to a complex pattern whereby social context and individual differences moderate the central release of oxytocin as well as moderate the effects that exogenous administration of oxytocin has on social behavior. In addition, it is becoming evident that measuring endogenous peripheral oxytocin levels is an informative tool. This is particularly so when oxytocin can be measured from non-invasively collected samples, such as in urine. Although it is still debated as to whether peripheral measures of oxytocin relate to central measures of oxytocin, anatomical and functional evidence indicate a link between the two. We argue that non-invasive measures of peripheral oxytocin hold several research and potential therapeutic advantages. Principally, study subjects can be sampled repeatedly in different social contexts where social history between interaction partners can be taken into account. Several hormones can be measured simultaneously allowing examination of the influence of oxytocin interactions with other hormones on motivational states. Valence of relationships as well as changes in relationship quality over time can be measured through endocrine responses. Also, the approach of identifying natural social contexts that are associated with endogenous oxytocin release offers the potential of behavioral therapy as an addition or alternative to chemical therapy in the field of mental health.

## Introduction

Oxytocin is known to facilitate affiliation, social bonding, and stress regulation. Recently, however, strong indicators have emerged that such beneficial effects of oxytocin may occur only under specific social circumstances (Bartz et al., 2011; Olff et al., 2013). Recent evidence also suggests that slight variation to social circumstances may impact on endogenous oxytocin *release* (Grewen et al., 2005; Zak et al., 2005; Seltzer et al., 2010; Kéri and Kiss, 2011). Such circumstances include, for example, the subjects' relationship to the interaction partner or whether trust is evoked instead of anxiety. In addition, the effects that oxytocin has and what triggers oxytocin release in a given social context can both vary depending on individual differences such as sex, age, early experience, psychiatric or psychological health (effects of oxytocin: Bartz et al., 2011; Macdonald and Feifel, 2013; Olff et al., 2013; release of oxytocin: Sanders et al., 1990; Turner et al., 1999). In this review, we further examine the extent to which the *release* of oxytocin and the *effects* of oxytocin vary according to specific social circumstances or individual differences. We are particularly interested in this question with respect to understanding the dynamic processes of relationship formation, maintenance, and degradation. We discuss the contribution that studies measuring endogenous peripheral oxytocin levels can offer to such questions.

Due to the anxiety-reducing and affiliative effects of oxytocin (Olff et al., 2013), much research effort goes toward the potential clinical use of oxytocin for ameliorating antisocial or isolating behavior, particularly within the context of mental health (Macdonald and Feifel, 2013). Of these studies, the focus has been almost exclusively on determining what oxytocin *does*, to whom and in what contexts (Bartz et al., 2011; Olff et al., 2013; Veening and Olivier, 2013 for reviews). In order to understand the interaction between the oxytocinergic system, social context and individual differences, it is also important to examine the other side of the system, what social behaviors and contexts *trigger the release* of endogenous oxytocin. Indeed, endogenous oxytocin may be an important regulator and a biomarker of subjects' social motivation toward, or perception of, a given social context (Bartz et al., 2011; Macdonald and Feifel, 2013).

Various theoretical approaches are available for mapping the complexity and variability within the oxytocinergic system. Comparative studies, for example, have shown that the distribution of oxytocin in the brain is well conserved across mammals. However, oxytocin *receptor* distributions are highly variable across species, and to some extent within species (Insel, 2010), suggesting considerable functional variation is likely between species (Goodson and Thompson, 2010) and within species (Insel, 2010). Comparative study can show the processes by which competing behavioral and physiological demands on common neural systems interact and constrain each other during evolution (Curley and Keverne, 2005; Goodson and Thompson, 2010). Within-species studies are already demonstrating considerable variation between individuals on the effects that oxytocin has on social behavior, as noted above (for reviews see Bartz et al., 2011; Olff et al., 2013).

An additional major source of variation in oxytocin reactivity to certain social contexts and across individuals is likely related to interactions between the oxytocinergic system and other hormonal and neurotransmitter systems (for reviews see Heinrichs et al., 2009; Soares et al., 2010; Neumann and Landgraf, 2012), such as with cortisol (see Szczepanska-Sadowska, 2008; Heinrichs et al., 2009), vasopressin (Neumann and Landgraf, 2012), estrogens (Amico et al., 1981; Zak and Fakhar, 2006), prolactin (Christensen et al., 2011), endorphins (Dunbar, 2010), testosterone (van Anders et al., 2011), dopaminergic and serotonergic systems (Skuse and Gallagher, 2009). Such interactions may account for different behavioral and motivational states associated with high oxytocin levels in both animal and human studies, for example, relaxed parent-infant interactions that engender affiliative and nurturant—“tend-and-befriend”—motivations, compared with in-group vs. out-group interactions that engender more affiliative and defensive—“tend-and-defend” motivations (Taylor et al., 2006; Campbell, 2008; Ross and Young, 2009; van Anders et al., 2011; De Dreu, 2012). How connections between different endocrine pathways influence engagement in particular social contexts is an emerging field, and one that is likely to be highly fruitful in determining variation in social motivation and perception as well as specific patterns of behavior.

In this review, we further examine under what contextual or individual circumstances endogenous peripheral oxytocin levels vary. In addition, we review suitable theoretical approaches to examine this variation. First, we briefly summarize the effects of oxytocin on social behavior, which have been covered in several recent and extensive reviews (Heinrichs et al., 2009; Ross and Young, 2009; Bartz et al., 2011; Churchland and Winkielman, 2012; De Dreu, 2012; Olff et al., 2013; Veening and Olivier, 2013). We then review known *triggers* of oxytocin release, to which there has been less focused attention. Research indicates that interactions of the oxytocinergic system with behavior, social environment, and endocrinological environment may be more nuanced in primates than rodents (Curley and Keverne, 2005). Thus, in terms of future directions, we discuss the strengths and weaknesses of the different approaches for examining more nuanced oxytocin-related interactions, and in particular emphasize the contribution of non-invasive approaches for measuring endogenous peripheral oxytocin.

## Effects of oxytocin on behavior and social context

Oxytocin has several classes of effects on behavior and social context. Oxytocin is known to play a key role in social bonding (Ross and Young, 2009), having both short-term and long-term effects (Winslow et al., 2003). Oxytocin also has strong anxiolytic—or stress-reducing effects (Churchland and Winkielman, 2012; Veening and Olivier, 2013). Furthermore, it facilitates social cognition, enhances social memory, social recognition and attention, and operates in the reward centers of the brain (Insel, 2010). Finally, in some studies oxytocin only increases positive perceptions or social motivations toward others (Chang et al., 2012; Shamay-Tsoory et al., 2013) and in other studies it also amplifies pre-existing social motivations or perceptions, whether positive or negative (De Dreu, 2012).

There is evidence that oxytocin has short-term effects on bond formation. Indeed, linking measures of central oxytocin to behavior in mammals has been critical for establishing the role of oxytocin beyond its central involvement in child birth and lactation (see Ross and Young, 2009 for a review), to that of a key component in bond formation between *mother and offspring* as well as in *pair bonds*. In terms of mothers bonding with their infants, when endogenous central oxytocin production is blocked, or when oxytocin receptors in the brain are blocked, bond formation is impaired (see Ross and Young for a review). In sheep, centrally administered oxytocin can induce maternal behavior in estrogen-primed, non-pregnant ewes (Kendrick et al., 1987). Vaginocervical stimulation, a known potent trigger of oxytocin release, can promote adoption in estrogen-primed ewes (Keverne et al., 1983). Oxytocin is also critical for inducing bonding from the infant toward the mother. For example, in contrast to wild-type mice pups, oxytocin receptor knock-out mice pups showed no preference for their mother over a novel female (Ross and Young, 2009). In terms of pair bonding, female prairie vole mate preferences can be formed by oxytocin infusion (Williams et al., 1994) and blocked by an oxytocin agonist (Insel and Hulihan, 1995).

There is evidence that social experience during childhood has long-term effects on the oxytocin system, impacting on mothering styles later in life. Mothering styles, for example, alter an offspring's oxytocin gene receptor (OXR) expression, which then remains unchanged into adulthood. Cross-fostered rats with foster mothers who licked and groomed them (LG) at low rates become low LG mothers. Whereas pups with high LG foster mothers became high LG mothers. Furthermore, high LG pups had higher OXR binding in the amygdala as adults (Francis et al., 1999, 2000). In addition, a single shot of oxytocin in pups shortly after birth was associated with increased participation in allo-parental care when they became adults (Bales et al., 2007). Also of note, in rhesus macaques, adolescents raised in a nursery by human caregivers had lower cerebrospinal fluid (CSF) oxytocin concentrations than those raised by their mother (Winslow et al., 2003). Similarly, in humans, women who experienced early childhood neglect or abuse had lower CSF oxytocin levels than those who did not report such experiences (Heim et al., 2008). In the latter two studies, what the effects of low CSF oxytocin levels might be for the subjects are not yet clear.

Oxytocin buffers the effects of stress (for recent reviews see Heinrichs et al., 2009; Olff et al., 2013). Oxytocin—and oxytocin-like neuropeptide—administration has long been known to decrease blood pressure in various species (Paton and Watson, 1912; Woodbury and Abreu, 1944). Intraperitoneally-administered oxytocin lowered cortisol release and increased wound-healing in paired hamsters, whereas a centrally-administered oxytocin agonist decreased wound-healing (Detillion et al., 2004). Men performing a stressful task showed the lowest plasma cortisol levels following both receiving 24 IU of intranasal oxytocin and social support from their best friend, compared with receiving a placebo or having no social support (Heinrichs et al., 2003). Also, Ditzen et al. (2009) showed that administering 40 IU of intranasal oxytocin increased positive communication during and decreased salivary cortisol after couple conflict in both men and women. Furthermore, Cardoso et al. (2013) showed that 24 IU but not 48 IU of inhaled oxytocin attenuated salivary cortisol levels during a subsequent physical stressor.

A further effect of oxytocin is in anxiety-reduction (see Churchland and Winkielman, 2012; Veening and Olivier, 2013 for reviews). Centrally-administered oxytocin reduced anxiety-related behavior in male mice during non-social behavioral tasks (Ring et al., 2006). Studies using inhaled oxytocin have demonstrated that exogenous oxytocin has anxiolytic potential, as when angry, happy or neutral faces were rapidly displayed in a computer screen, humans, using a joystick, were more likely to “approach” rather than “retreat” from angry faces following 24 IU of oxytocin (Radke et al., 2013). It has been considerably debated to what degree oxytocin actually induces changes in social perception or motivation toward being more prosocial, as detailed below, or rather that changes are a by-product of oxytocin reducing social fear and anxiety (see Churchland and Winkielman, 2012). It has also been argued that these purported effects of oxytocin do not have to be mutually exclusive but may operate in tandem (Bartz et al., 2011).

Oxytocin also effects social cognition, particularly in capacities that are likely to facilitate social interaction. It increases gaze to faces and improves social recognition (see Guastella and MacLeod, 2012 for review). It also activates social memory areas in the brain (Dantzer et al., 1987; Ferguson et al., 2000). Exogenous oxytocin may also change individuals' perceptions or social motivation toward others or within a particular social context to become more affiliative, attentive, prosocial, or empathetic. In rhesus macaques, for example, Chang et al. (2012) found that subjects that engaged in a food sharing task showed first greater selfish choices and after 2 h greater prosocial choices after inhaling oxytocin. Parr et al. (2013) found that inhaled oxytocin affected the monkeys' social perceptions by reducing their attention to negative but not neutral facial expressions. In another study, administering oxytocin into peripheral blood in meerkats induced a whole suite of cooperative behaviors including pup-feeding, guarding, digging and decreased aggression (Madden and Clutton-Brock, 2011), suggestive of enhanced prosocial motivation. Furthermore, using an in-group vs. out-group paradigm, Shamay-Tsoory et al. (2013) showed that for Israeli Jewish subjects, inhaled oxytocin increased empathy toward potential out-group (Palestinian Arab) members but not to potential in-group members where empathy levels were already high.

As a final effect of oxytocin, it is thought that under certain circumstances, oxytocin may activate a positive feedback system, such that oxytocin may trigger a behavior or motivation, or vice versa. Then, the behavior or motivation may trigger further oxytocin release, which may again initiate more of the behavior (Uvnäs-Moberg, 1998; De Dreu, 2012). There are indications for this in the context of labor during child birth (Russell et al., 2003), and this is hypothesized, for example, in the context of affiliation (Uvnäs-Moberg, 1998) and cooperation (see De Dreu, 2012). However, a critical aspect of examining a positive feedback system will be to measure endogenous oxytocin levels, not only to administer oxytocin.

## Behaviors and social contexts that trigger the release of oxytocin

Behaviors identified as triggering the release of oxytocin can be broadly classified into two groups: first, sexual and intimate behaviors, and second, stressful events. Mating-induced effects, specifically vaginocervical stimulation in rodents (Sansone et al., 2002) and sheep (Kendrick et al., 1986), and orgasm in men and women (Ogawa et al., 1980; Carmichael et al., 1987) increase plasma oxytocin levels, whilst vaginocervical stimulation in prairie voles increases oxytocin levels in the brain (Ross et al., 2009a,b). In terms of mother-infant behavior, Juszczak and Stempniak (1997) found that suckling caused the release of stored oxytocin from the neurohypophysis into the blood of female rats. Likewise, others have found that suckling or nipple stimulation increases plasma oxytocin in rats (Neumann et al., 2000) as well as in full-term pregnant women (Christensson et al., 1989). There is also evidence that touch alone, outside of sexual or parental relationships, triggers oxytocin release. Stimulation of peripheral afferent nerves with pleasant (stroking) or unpleasant (foot pinching) touch rapidly increased plasma oxytocin levels in rats, as did direct electrical stimulation of the vagal and sciatic nerves (Stock and Uvnäs-Moberg, 1988). In humans, however, only affiliative touch has been associated with increased salivary oxytocin (Holt-Lunstad et al., 2008) and plasma oxytocin levels (Turner et al., 1999). It is not clear if, like with rodents, unpleasant touch in humans also releases oxytocin.

In terms of stress-related oxytocin triggers, both non-social and social stressors are associated with oxytocin release (DeVries et al., 2003; Bartz and Hollander, 2006; Table 1). Socially isolated rats receiving stressors of restraint and ether, but not cold, showed raised plasma oxytocin levels (Gibbs, 1984), indicating that some, but not all, non-social stressors precipitate peripheral oxytocin release. In humans, however, non-social stress alone does not always trigger the release of oxytocin. Seltzer et al. (2010) showed that urinary oxytocin levels were only raised following a stressor when the stressor was followed by a comforting vocal or physical contact with subjects' mothers. In the control condition, where exposure to a stressor was followed by subjects watching a film on their own, elevated salivary cortisol but not urinary oxytocin levels were detected. In another study, oxytocin was released following a non-social stressor, but only for certain types of individual. Indeed, high emotionality—or anxious—women, but not low emotionality women or men, showed raised plasma oxytocin after hearing noise through headphones that they could not control compared to noise that they could control, whilst completing a memory task (Sanders et al., 1990). Whilst the relationship between stress and oxytocin may be straightforward in rodents, the relationship may be more dependent on social context in humans.

**Table 1:** **The relationship between specific social behaviors and endogenous peripheral oxytocin levels compared with baseline levels**.

**Behavior**	**Conditions**	**Interaction partner**	**Species**	**Δ OT**	**Body fluid**	**Sex**	**Study**
**POSITIVE (DYADIC^+^) TACTILE STIMULATION**							
Genital	Vaginocervical stimulation	Experimenter	Rat	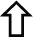	Blood	F	Sansone et al., 2002
		Experimenter	Sheep	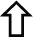	Blood	F	Kendrick et al., 1986
	Orgasm	Self	Human	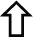	Blood	M	Ogawa et al., 1980
		Self	Human	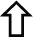	Blood	F, M	Carmichael et al., 1987
Nipple	Mother suckling	Offspring	Rat	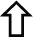	Blood	F	Juszczak and Stempniak, 1997
		Offspring	Rat	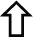	Blood	F	Neumann et al., 2000
		Offspring	Dog	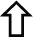	Blood	F	Uvnäs-Moberg et al., 1985
		Offspring	Pig	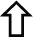	Blood	F	Uvnäs-Moberg et al., 1985
	Rates of mother suckling and grooming	Offspring	Rhesus macaque	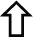	Blood	F	Maestripieri et al., 2009
	Nipple stimulation	Self	Human	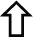	Blood	F	Christensson et al., 1989
Body	Stroking	Experimenter	Rat	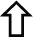	Blood	M	Stock and Uvnäs-Moberg, 1988
	Pinching (negative)	Experimenter	Rat	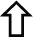	Blood	M	
	Stroking	Owner	Dog	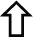	Blood	M	Handlin et al., 2011
	Stroking	Known person	Dog	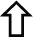	Urine	F, M	Mitsui et al., 2011
	Rates of grooming and sexual contact	Mate	Tamarin monkey	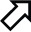	Urine	F, M	Snowdon et al., 2010
	Grooming						
	- donors and receivers	Bond partner	Chimpanzee	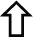	Urine	F, M	Crockford et al., 2013
		Non-bond partner		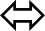	Urine	F, M	Crockford et al., 2013
	Massage	Stranger	Human	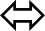	Blood	F, M	Morhenn et al., 2008
	- no relationship anxiety	Stranger	Human	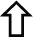	Blood	F	Turner et al., 1999
	- relationship anxiety	Stranger	Human	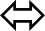	Blood	F	Turner et al., 1999
	- after weeks of massage course	Spouse	Human	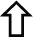	Saliva	F, M	Holt-Lunstad et al., 2008
	Warm contact						
	- rating of partner support	Partner	Human	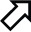	Blood	F, M	Grewen et al., 2005
	Massage then trust game	Stranger	Human	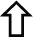	Blood	F, M	Morhenn et al., 2008
	Play						
	- child	Mother	Human	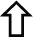	Urine	F, M	Fries et al., 2005
	- child	Stranger	Human	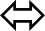	Urine	F, M	Fries et al., 2005
	- neglected child	Foster carer	Human	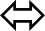	Urine	F, M	Fries et al., 2005
	- neglected child	Stranger	Human	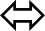	Urine	F, M	Fries et al., 2005
	- mother	Own child	Human	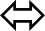	Urine	F	Bick and Dozier, 2010
	- mother	Stranger child	Human	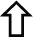	Urine	F	Bick and Dozier, 2010
	Bondedness						
	- mothers	Own infant	Human	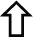	Blood	F	Feldman et al., 2007
	Synchrony						
	- fathers	Own infant	Human	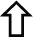	Blood	M	Gordon et al., 2010
**FOOD RELATED**							
	Being fed	Unspecified	Dog	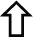	Blood	F	Uvnäs-Moberg et al., 1985
	Being fed	Known person	Dog	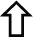	Urine	F, M	Mitsui et al., 2011
	Being fed	Unspecified	Pig	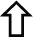	Blood	F	Uvnäs-Moberg et al., 1985
	Food sharing (control: self-feeding)						
	- donors and receivers	Bond and non-bond partners	Chimpanzee	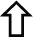	Urine	F, M	Wittig et al., 2014
**DYADIC WITHOUT TACTILE STIMULATION OR CONTACT**							
	Trust Game	Stranger	Human	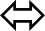	Blood	F, M	Morhenn et al., 2008
	- receiving trusting offer	Anonymous	Human	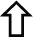	Blood	F, M	Zak et al., 2005
	- receiving automated offer	Computer	Human	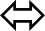	Blood	F, M	Zak et al., 2005
	- share secret	Experimenter	Human	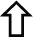	Blood	F, M	Kéri and Kiss, 2011
	- share neutral information	Experimenter	Human	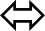	Blood	F, M	Kéri and Kiss, 2011
	Social stressor	Experimenter	Human	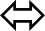	Urine	F	Seltzer et al., 2010
	- then neutral activity	TV	Human	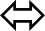	Urine	F	Seltzer et al., 2010
	- then tactile comfort*	Mother	Human	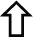	Urine	F	Seltzer et al., 2010
	- then vocal comfort	Mother	Human	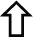	Urine	F	Seltzer et al., 2010
**EXERCISING**							
	Commanded to trot	Known human	Dog	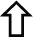	Urine	F, M	Mitsui et al., 2011
**NON-SOCIAL, NON-TACTILE STRESSOR**							
	Restraint	–	Rat	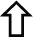	Blood	M	Gibbs, 1984
	Ether		Rat	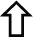	Blood	M	Gibbs, 1984
	Cold		Rat	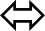	Blood	M	Gibbs, 1984
	Hearing uncontrollable noise through headphones						
	- not highly emotional	Experimenter	Human	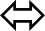	Blood	F, M	Sanders et al., 1990
	- highly emotional	Experimenter	Human	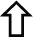	Blood	F, M	Sanders et al., 1990

*This table does not attempt to be exhaustive but rather to provide an overview of the range of studies conducted, specifically the ones mentioned in this review. Δ OT: change in oxytocin levels after engaging in the behavior compared with baseline condition.* 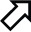
*: positive correlation.* 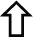
*: oxytocin levels were higher or increased.* 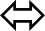: oxytocin levels were not different. ^+^“Dyadic” indicates that the behavior has principally evolved as a dyadic behavior even if it was not dyadic in the cited studies. ^*^An exception for this section, involving tactile contact.}

## The role of oxytocin in social bonding between friends

The above section shows that the main known triggers of oxytocin release are related to sexual or maternal behaviors. More recent studies with humans show that oxytocin release in association with affiliative touch and other contexts does occur but is subject to changes in social circumstances and individual differences, the particular effects of which are not yet well established. Also, for humans stressors alone do not readily trigger oxytocin release (see Table 1). In some large social mammals, maintaining “friendships” (defined here as “enduring close social bonds”) outside of mating or maternal relationships carries fitness benefits by enhancing own and offspring survival (Seyfarth and Cheney, 2012). Individuals who maintain bonds between same-sex adults compared with those who do not, have more offspring, live longer and are healthier (monkeys: Schülke et al., 2010; feral horses: Cameron et al., 2009; humans: Holt-Lunstad et al., 2010). In some cases these beneficial bonded relationships are with kin, such as between sisters (Silk et al., 2003, 2010), but in some cases they are between non-kin (Cameron et al., 2009; Schülke et al., 2010; humans: Holt-Lunstad et al., 2010). This begs the question, how are close social bonds maintained between friends, in the absence of potent oxytocin releaser behaviors such as lactation and orgasm? There is some evidence that oxytocin release is more likely during interactions with certain individuals, such as bond partners (Seltzer et al., 2010; Crockford et al., 2013). It may be that oxytocin released during interactions with bond partners enhances the social memory of those interactions (see Choleris et al., 2009; Ross and Young, 2009; Brent et al., 2013 for reviews), reinforcing partner-specific preferences. For bond formation, and particularly bond maintenance to be successful, the oxytocin system may link in to other mechanisms, for example, to connect with the neural reward system. In monogamous but not polygamous vole species, oxytocin receptors are found in the nucleus accumbens, a neural center associated with reward and reinforcement (Liu and Wang, 2003). Behaviors or social contexts associated with oxytocin may be influenced by an associated sense of reward and may thus, be more likely to occur again (Broad et al., 2006). This is potentially a crucial feature of social bond formation and maintenance (Broad et al., 2006; Insel, 2010).

Thus, there are three aspects likely to be of particular importance when trying to understand the dynamic processes and trajectory of social interactions and relationships. Oxytocin is an enhancer of certain aspects of social cognition, such as social attention, memory, and prosocial motivation. It is associated with neural reward and reinforcement. In combination, these neural effects are likely to selectively increase the probability of repeated, affiliative social interactions with particular individuals, such as bond partners. The chances of such an effect occurring increase when considering a third aspect, that there may be a positive feedback system between oxytocin release and certain behaviors or motivations. Whether positive feedback might happen under some or all conditions of initial oxytocin release is not known. Likewise, what precipitates cessation of such a positive feedback loop is also unknown. Nonetheless, these features of the oxytocin system may be contributors to both bond formation and bond maintenance.

## The strengths and weaknesses of examining exogenous vs. endogenous peripheral oxytocin with associated behavior

A significant limitation of studying central oxytocin is that it requires direct access into the brain, often by sacrificing study subjects. This is an acceptable practice for work with rodents. It is of course not possible when studying how oxytocin works in larger mammals, particularly in primates, whether human or otherwise. fMRI techniques are also not yet at a point where study subjects can be anything other than restrained during the examination (e.g., Bethlehem et al., 2012; Rilling et al., 2012), rendering them impractical for examining natural triggers or effects of oxytocin on animals and humans when engaged in contexts representative of natural social and environmental life. A necessary feature of researching the changes in relationships over time is being able to conduct repeated sampling from repeated interactions over time, again difficult to conduct with current invasive methods.

Thus, several additional, less invasive approaches for examining the interaction between social behavior and oxytocin have been established. The first approach is to administer oxytocin into the peripheral system and examine effects on subsequent behavior. The second approach is to observe or manipulate behavior and then measure subsequent endogenous levels of peripheral oxytocin. However, both of these less invasive approaches have been criticized (see Macdonald and Feifel, 2013 for review). Exogenous oxytocin studies are introduced briefly below. Studies that measure endogenous oxytocin, being the main focus of this paper, are explained in detail. We examine the gains and limitations of each approach.

### Exogenous peripheral oxytocin studies

Although it has not been clear if or how inhaled oxytocin might affect the brain (See Veening and Olivier, 2013 for in-depth review), many studies have shown correlations between inhaled oxytocin and predicted behavior. More recently, Neumann et al. (2013) in mice and rats, and Chang et al. (2012) in rhesus monkeys showed that inhaled oxytocin arrives in the behaviorally-relevant parts of the brain and in the CSF, respectively, although the exact route remains elusive. Furthermore, Neumann et al. (2013) demonstrated that following inhaled oxytocin, plasma levels of oxytocin paralleled central levels.

The considerable recent research investment in oxytocin-associated studies is linked to its perceived potential to be a modern day panacea to resolve many social problems, especially those related to ameliorating antisocial or isolating behavior. Many studies show ameliorating effects of oxytocin on psychological stress and psychiatric disorders (Striepens et al., 2011; Macdonald and Feifel, 2013). However, a number of studies in healthy people, as well as in patients with psychological or psychiatric disorders show that administration of oxytocin leads to negative not positive social effects (Epperson et al., 1996; Bartz et al., 2011; Feifel, 2011; Mah et al., 2012), suggesting that exogenous oxytocin does not always lead to increased affiliation. Some of these effects may be accounted for by dosage issues mentioned below. Also, how much these differences are related to interactions between oxytocin and other hormonal interactions, neural connectivity, genetic expression, developmental history and social context remains to be seen.

In a recent and detailed review, Olff et al. (2013) suggest that oxytocin may augment pre-existing social perceptions or the valence of specific relationships, whether positive or negative. This is especially evident in studies relating to behavioral and physiological differences with in-group compared to out-group contexts, where oxytocin typically increases cooperation toward those perceived as being in-group members but simultaneously decreases cooperation and increases negative attitudes toward out-group members (see De Dreu, 2012 for a review). A notable exception comes from Shamay-Tsoory et al. (2013) showing that under some circumstances administered oxytocin may alter the valence of out-group perception from negative to more positive.

One way to disentangle contradictory results from studies examining interactions between oxytocin, behavior, other hormones and genes is to examine similar social contexts and hormones in closely-related animal models. Here, using free-living animals may be critical, however. Given the apparent sensitivity of the oxytocinergic system as well as of social and emotional development to psychological trauma during ontogeny (Francis et al., 1999, 2000; Winslow et al., 2003; Heim et al., 2008), careful examination of life-histories should be considered before embarking on oxytocin-related studies with captive animals. Captive apes, for example, have often experienced early trauma, lived in isolation or have suffered abuse. Those that have, often show disturbed behavior and social interactions compared to those who have not (Ferdowsian et al., 2011; Clay and de Waal, 2013). Likewise, in addition to laboratory experiments, examining the effects of administered oxytocin in healthy humans as they interact in more natural contexts with known social partners is likely to help clarify how contextual variation and individual differences alter relationship dynamics over time (Seltzer et al., 2010; Feldman, 2012).

The current limitations of the inhaled oxytocin approach are that as yet, dosage has been hard to control and indeed there is no consensus on optimal doses for use in studies (Cardoso et al., 2013). Studies that have controlled for dose show important dose-dependent variation on physiology (Cardoso et al., 2013) or social cognition (Goldman et al., 2011; Hall et al., 2012). Middle doses (20–24 IU) have the greatest positive effects, whereas low (10 IU) or high doses (48 IU) show blunted effects (Goldman et al., 2011; Hall et al., 2012; Cardoso et al., 2013). It is possible that some studies with contradictory results may arise from administering particularly high oxytocin doses. There is some evidence that with high concentrations in the brain, oxytocin begins to occupy structurally related arginine-vasopressin receptors. Given that vasopressin has opposing effects on behavior to oxytocin, this could account for contradictory results (Legros, 2001; Olff et al., 2013). Likewise, determining how applied doses may relate to endogenous oxytocin levels and their effects has not been addressable, partly because few studies measure endogenous oxytocin levels in conjunction with oxytocin administration (review: Bartz et al., 2011; Olff et al., 2013).

### Endogenous peripheral oxytocin studies

Apart from administration of oxytocin into the periphery, a second approach for examining peripheral oxytocin has been to measure endogenous levels of peripheral oxytocin from plasma, urine or saliva following involvement in a certain social context or behavior. Whilst studies examining exogenous oxytocin studies are critical for determining the *effects* of oxytocin on other physiological processes as well as on behavior and cognition, they tell us little about what factors precipitate oxytocin *release*. As with examining the effects of exogenous oxytocin, examination of endogenous oxytocin levels indicate that factors that precipitate oxytocin release vary depending on social context as well as on individual differences (Table 1).

Many studies now examine peripheral measures of oxytocin in relation to pathological conditions in humans (e.g., depression, autism, schizophrenia) (for review see Macdonald and Feifel, 2013). Some studies link peripheral oxytocin measures to oxytocin receptor genotype findings, for example, that negative correlations between salivary oxytocin and chronically depressed mothers, their husbands, and children are more prevalent in people with certain genotypes (Apter-Levy et al., 2013; Feldman et al., 2013). However, it is beyond the scope of this review to examine the literature researching the link between oxytocin function and psychiatric disorders. Here, we focus on what peripheral oxytocin measures can tell us about natural or normal biological systems and evolutionary traits.

#### Tracking relationship valence between interaction partners using endogenous oxytocin measures

Several studies show that peripheral oxytocin levels provide an objective measure of relationship strength or quality in both human and non-human primates and therefore, might be useful in animal behavior and cognition studies, as well as in human research or therapy validation (Table 1). In primates, a study in pair-bonded tamarins examining behavioral correlates over time with repeated samples of urinary oxytocin, showed that pairs that had a closer bond, defined as engaging in higher rather than lower rates of affiliative and sexual behaviors over several weeks also had higher urinary oxytocin concentrations during the same time period (Snowdon et al., 2010). These results parallel those of an exogenous oxytocin study on marmosets where daily treatment of 50 μg for 30 days facilitated pair-bond formation (Smith et al., 2010). Also, composite measures of mothers' suckling and grooming rates with offspring collected over a 3-month period correlated positively with one-off plasma oxytocin samples in rhesus macaques (Maestripieri et al., 2009). Free-ranging male and female chimpanzees had higher urinary oxytocin levels following at least 10 min of grooming with a bond partner compared with resting controls. However, urinary oxytocin levels were not different from resting controls when grooming occurred with a non-bonded partner (Crockford et al., 2013).

The finding in chimpanzees that a close social relationship *in combination with* affiliative touch was associated with high urinary oxytocin levels concurs with studies on humans showing that prolonged affiliative touch alone is not always an efficient way to increase endogenous oxytocin levels. Morhenn et al. (2008) showed that in both men and women, plasma oxytocin levels were raised following a combination of 15 min of massage followed by a monetary trust game. Plasma oxytocin levels, however, were not raised following either a massage or trust game alone. In a study with women, Turner et al. (1999) showed that a raise in plasma oxytocin following gentle massage occurred only in women who did not report relationship anxiety. Also, salivary oxytocin levels increased after several weeks of long-term couples practicing gentle massage intervention with each other (Holt-Lunstad et al., 2008). Taken together, these studies suggest that at least for humans and chimpanzees, a complex set of social factors influence whether affiliative touch triggers endogenous oxytocin release. This may not be the same for rats, where both positive and negative touch resulted in raised plasma oxytocin levels (Stock and Uvnäs-Moberg, 1988). See Table 1.

In other human studies, Fries et al. (2005) showed that children who played with their principle carer rather than with strangers showed higher urinary oxytocin levels whereas orphans who had suffered severe neglect showed no change in urinary oxytocin levels across conditions. In contrast, however, Bick and Dozier (2010) showed that mothers had higher urinary oxytocin after playing with a stranger child compared with their own child. Other studies show that high plasma oxytocin levels correlated postively with the level of mother-infant bondedness (Feldman et al., 2007) and father-infant synchrony (Gordon et al., 2010). Grewen et al. (2005) found positive correlations between personal ratings of partner support and plasma oxytocin levels both in baseline conditions and following 10 min of warm contact. The studies in both human and non-human primates suggest that endogenous oxytocin measures may act as a biomarker for the valence and strength of the relationship between interaction partners.

In conclusion, most of the studies on human and non-human primates suggest that the oxytocin release associated with affiliative or trusting contact with a bonded other is a result of a psychological component, such as an affiliative or trusting perception or motivation toward a specific other, within the context of affiliative touch. However, to determine how much oxytocin release is dependent on an individuals' perception or motivation rather than the physical properties of the social interaction (how gentle or intimate the affiliative touch is), careful assessment is needed of both the physical properties of the interaction as well as subjects' perceptions and motivation prior to and following the social interaction. That oxytocin release may depend more on an individuals' social perception of an interaction than on the properties of the interaction itself mirrors other studies on human and non-human primates which suggest that inhaled oxytocin changes individuals' perception or motivation toward a social context (Chang et al., 2012; Parr et al., 2013, for reviews see: Bartz et al., 2011; Olff et al., 2013). Whether such a mirroring effect again points to a positive feedback system between oxytocin release and an interaction of social context with the perception of that context, remains to be seen.

The finding that oxytocin release is associated with an interaction between affiliative touch and a psychological component related to the touch context suggest that endogenous oxytocin can be used to track valence changes in relationships over time, in both males and females. This could be useful in animal studies to provide physiological validation of behavioral observations. It could also be useful in human relationship therapy as a biomarker of therapy progress. The oxytocin system of children who have suffered severe neglect appeared less responsive to contact with bond partners (Fries et al., 2005). Whether this low reactivity reflects the childrens' feelings of emotional engagement with their carers remains to be examined. In some studies, both baseline measures of endogenous oxytocin and oxytocin responsiveness to particular social circumstances correlate with subjective ratings of feelings and emotional states (e.g., Grewen et al., 2005) and thus may act as an objective measure of capacity to bond as well as progress in bonding therapy.

#### Endogenous oxytocin varies depending on cooperative behavior and cooperation partner

Some cooperative behavior is associated with the social motivation to trust and the perception that others are trustworthy. Zak et al. (2005) found that individuals receiving trusting money offers had higher plasma oxytocin levels 2 min later than those who did not. Likewise, Kéri and Kiss (2011) tested subjects engaging with an experimenter in a trust task (sharing a personal secret) and found that those who exchanged a secret with the experimenter as opposed to exchanging neutral information or engaging in a (non-social) stress condition, had raised plasma oxytocin immediately afterwards.

De Dreu (2012) underlines the importance of examining oxytocin in a broader range of behavioral contexts that also include interactions between individuals with a shared interaction history, whether of cooperation or potential conflict. Indeed, Declerck et al. (2013) have shown that for a subset of human subjects with greater prosocial as opposed to selfish tendencies, cooperative choices in a one-shot prisoner's dilemma game were more positively influenced by brief prior contact with their game partner than by 24 IU of intranasal oxytocin. Although endogenous measures of oxytocin were not taken in this study, the results suggest that, at least for certain personality types, even brief prior contact can have a significant impact on cooperative outcome.

Two studies examining cooperative acts and oxytocin conducted on wild chimpanzees in the Budongo Forest, Uganda show a more complex relationship between cooperative acts and shared interaction history (Crockford et al., 2013; Wittig et al., 2014). Two cooperative behaviors, allo-grooming and food sharing, were associated with significantly higher urinary oxytocin levels than non-cooperative behaviors, feeding in the presence of others without sharing, or resting. In the grooming context, only subjects who groomed with bond partners, as opposed to known but neutral others, had higher urinary oxytocin levels than in the resting condition. In the food sharing context, however, urinary oxytocin levels were high irrespective of the valence of the relationship between sharing partners. The effects were similar whether subjects were donors or recipients of grooming or food sharing, and whether donors and receivers were male or female. The results suggest that engaging in certain, but not all, cooperative acts with a bond partner results in a stronger positive correlation with urinary oxytocin levels than cooperating with mere acquaintances.

There are further implications of the chimpanzee studies. There is some indication that variation in endogenous levels of oxytocin may be an indicator not only of the strength of a relationship but also of the *bonding value* of a particular behavior or a social context. Both cooperative acts, that is food sharing and high rates of grooming interactions, are considered key indicators of enduring, bonded relationships in chimpanzees (Langergraber et al., 2007; Mitani, 2009). Thus, it is interesting that we found variation in chimpanzee urinary oxytocin levels in relation to the cooperative context. Food sharing, a relatively rare behavior, was associated with significantly higher urinary oxytocin levels than grooming with bond partners (Wittig et al., 2014). Further testing is required, however, in both the chimpanzee and the human studies mentioned in the previous section, to determine if significantly higher urinary oxytocin levels are indicative of greater subsequent bonding or cooperative exchange than lower oxytocin levels.

The result that not self-feeding but food sharing *per se* is associated with high urinary oxytocin levels suggests caution is needed when using feeding paradigms in oxytocin studies. Uvnäs-Moberg et al. (1985), for example, tested dogs and pigs in suckling and feeding contexts. Using a catheter attached to the subjects' leg they could obtain plasma samples every 30 s. They found increased plasma oxytocin in mothers during suckling, as well as when mothers and adult males themselves fed. Interestingly the study also showed an anticipatory raise in oxytocin up to 2 min prior to receiving food. Another study with dogs also showed a rise in oxytocin following feeding (Mitsui et al., 2011). In both studies however, the influence of the animal carer providing the food is not considered. Indeed, a subsequent study showed that 3 min after dog owners stroked their dogs, dog plasma oxytocin levels had increased (Handlin et al., 2011). It may be that in the feeding studies, social interaction with feeding, rather than feeding itself, was the oxytocin trigger. This would need to be verified in further studies.

Taken together studies on relationship quality, cooperative acts and endogenous oxytocin suggest complex, nuanced interactions between endogenous oxytocin, social context, social motivation and perception. Participants' shared history with the interaction partner has clear, and to some extent predictable associations with endogenous oxytocin levels. Some cooperative acts are associated with higher oxytocin levels than others, such as food sharing compared to grooming. Whether higher endogenous oxytocin levels following participation in certain cooperative acts compared to others indicates that they have greater bonding potential remains to be tested.

#### Determining multi-hormonal profiles for differing social relationships or cooperative contexts

A potential but as yet little used role for measuring endogenous hormone levels would be to examine links between complex behavior and motivation, such as, do cooperative tasks that require different motivational states, such as “tend-and befriend” compared with “tend-and-defend” motivations, have predictable hormonal interaction patterns (Taylor, 2006; Campbell, 2008; van Anders et al., 2011; De Dreu, 2012)? “Tend-and befriend” has been defined as nurturant behavior combined with the motivation to create and maintain a social network (Taylor, 2006). “Tend-and-defend” has been defined as the coinciding motivations to trust and cooperate with the in-group but engage in defensive behavior toward the out-group (De Dreu et al., 2010). Presumably the answer to the above question depends on what simultaneous physiological systems, whether hormones or neurotransmitters, are activated (for reviews see Skuse and Gallagher, 2009; Heinrichs et al., 2009; Neumann and Landgraf, 2012). In chimpanzees, hormonal interaction patterns are likely to differ depending on the cooperative task. In the context of food sharing in wild chimpanzees, urine samples following naturally-occurring food sharing events had low testosterone levels (Sobolewski et al., 2012) and high oxytocin levels (Wittig et al., 2014). A different pattern between these two hormones might be expected in in-group vs. out-group cooperative contexts. In wild chimpanzees, for example, territories require cooperative defense at a group level in the form of territorial boundary patrols. Sobolewski et al. (2012) showed that urinary testosterone was significantly higher following territorial boundary patrols compared with before patrols. Since intense grooming often occurs immediately prior to patrols, it might be expected that oxytocin, like testosterone, would also be high following patrols. Taken together, these studies suggest that high oxytocin and low testosterone might induce gentle affiliation, or nurturant behavior (van Anders et al., 2011)—a similar pattern as is expected for new human fathers (Storey et al., 2000; Gordon et al., 2010). In contrast, high oxytocin and high testosterone might induce loyal comradeship required for dangerous or risky group action that individuals are less likely to engage in alone (De Dreu, 2012), such as dare-devil acts or warfare.

When considering the oxytocinergic and the hypothalamic-pituitary-adrenocortical axis stress systems, different relative plasma concentrations of oxytocin and cortisol are evident depending on different social contexts and environmental influences. After an acute stressor, oxytocin may act as a buffer against stress (Heinrichs et al., 2009). In this case, following a stressor, endogenous peripheral hormone measures would be expected to show raised oxytocin but reduced cortisol concentrations. Seltzer et al. (2010) have shown, however, that for human children, after being exposed to an acute stressor, comforting physical or vocal contact with their mother increased urinary oxytocin levels and decreased salivary cortisol levels more quickly than in control conditions with no mother contact. This suggests that, at least in certain contexts for certain individuals, affiliative social contact may be required to trigger oxytocin release after an acute stressor. In contrast, a different pattern of endogenous cortisol and oxytocin levels may be apparent in praire voles subjected to a non-social stressor (cage flooding) whilst in a normal social context (being with their cage mates). Yee et al. (cited in Olff et al., 2013) showed that female voles pretreated with peripheral oxytocin had subsequent raised plasma oxytocin levels 95 min later. In the condition of oxytocin administration before the stressor, heart rate was lower than in the non-oxytocin condition. In contrast to other studies, this study also found a trend toward raised, rather than lowered cortisol. The interpretation is that maintaining higher cortisol levels could amount to maintaining vigilance, critical in a natural setting when facing a danger. Simultaneously maintaining higher oxytocin levels could result in increased social contact rather than promoting dispersal. A third hormonal interaction pattern for oxytocin and cortisol is shown by two other studies. Turner et al. (1999) and Taylor et al. (2006) found that older women who simultaneously maintained high baseline levels of plasma oxytocin and cortisol also had chronic relationship anxiety. Whether the pattern of simultaneously maintaining both high oxytocin and high cortisol levels in a chronic context (Turner et al., 1999; Taylor et al., 2006) shares any functional parallel with the similar pattern observed in a short-term context in the prairie voles, remains to be tested. For example, maintaining vigilance might be related to the threat of a physical stressor in the case of the praire voles, and a social stressor in the case of the older women. Finding that predictable patterns in endogenous hormonal sampling of several hormones simultaneously are dependent on social context interactions would prove highly illuminating in understanding underlying social motivations, perceptions and actions, as suggested by multi-hormone models (e.g., van Anders et al., 2011).

The interrelationship between oxytocin and cortisol raises further questions. As stated above, oxytocin seems to act as a buffer against stress. In some contexts in humans, however, its release seems more likely when a stressor is followed by affiliative contact (e.g., Seltzer et al., 2010). Might post-stressor oxytocin priming increase the likelihood of initiating affiliative social interactions that further buffer the effects of stress (Taylor, 2006)? Might the phenomenon of rapid and intense bonding between people during major stressors, people's first day at school or college or during major disasters such as train crashes, be accounted for by such mechanisms? Also, might people who can rapidly buffer stress through affiliative social contact be later less vulnerable to negative moods, or following major stressors, to post-traumatic stress disorders (see Bradley et al., 2013)? Clearly, however, stress does not always lead to affiliation, for example, in situations where the stressor was a conflict, one conflict can lead to another. Are such outcomes more likely in scenarios when oxytocin is not released following a stressor? Perhaps oxytocin is released but engenders a different emotional state, as shown in “tend-and-defend” in-group vs. out-group studies (De Dreu, 2012). Studies examining interactions of more than one hormone within particular social contexts could address such questions (van Anders et al., 2011; Neumann and Landgraf, 2012).

A potential problem for endogenous peripheral measures of oxytocin is, given both stress and affiliation are triggers of oxytocin release, can one be sure when measuring endogenous oxytocin whether it was released due to stress or affiliation? Interestingly, in a study in humans using an acute stressor (Seltzer et al., 2010), there was no signal that urinary oxytocin was raised or higher than baseline, unless there was subsequent affiliation or cooperation. Indeed, although rodent studies show oxytocin release in response to non-social stressors (e.g., Wotjak et al., 1998), few human—or non-human primate—studies have done so (see Sanders et al., 1990).

#### The limitations of the endogenous oxytocin approach

It is still debated if or how peripheral measures of oxytocin relate to central measures of oxytocin and therefore, whether measurements of peripheral oxytocin levels hold biological validity (see Macdonald and Feifel, 2013 for review of both sides of the debate). However, there is some evidence from both anatomical and functional studies showing a correlation between central and peripheral oxytocin release. From the anatomical side, two independent studies identified concomitant axonal projections from magnocellular neurons to various extra-hypothalamic brain regions and to the posterior pituitary. Stimulation of axonal projections led to release of oxytocin into the brain and the periphery (Ross et al., 2009a,b; Knobloch et al., 2012) resulting in expected changes to rat behavior (Knobloch et al., 2012). From the functional side, Wotjak et al. (1998) demonstrated that peripheral and central concentrations of oxytocin are simultaneously raised during forced swimming stress, suggesting a coordinated release of oxytocin into central and peripheral areas.

Most studies that link hormone levels with behavioral events demonstrate that oxytocin levels concur with predicted behavioral functions. In independent studies in rodents and sheep, contexts or behaviors shown to have central oxytocin involvement also show positive increases in plasma oxytocin levels (rodents: Ogawa et al., 1980; Carmichael et al., 1987; Stock and Uvnäs-Moberg, 1988; Juszczak and Stempniak, 1997; Neumann et al., 2000; sheep: Keverne et al., 1983; Kendrick et al., 1986). Congruent patterns are observed when linking social behaviors to endogenous measures of oxytocin in plasma, urine or saliva in non-human primates (Seltzer and Ziegler, 2007; Maestripieri et al., 2009; Snowdon et al., 2010; Crockford et al., 2013) and humans (Christensson et al., 1989; Holt-Lunstad et al., 2008; Morhenn et al., 2008; Gordon et al., 2010).

Several biological validations of oxytocin assays have been completed (see Macdonald and Feifel, 2013 for review). In tamarins, for example, Snowdon et al. (2010) implanted three males with an estradiol pellet (1.5 mg continuous release over 30 days) and collected urine samples before and during the period of the implant. Estradiol increased significantly in all three males (*t* = 3.64, *P* = 0.04) ensuring the effectiveness of the pellets in raising estradiol. Male oxytocin response varied substantially, but two of the three males showed a large increase in oxytocin following the estradiol elevation (mean pre *OT* = 17 ± 5 pg/mg Cr, mean post *OT* = 100 ± 15 pg/mg Cr). These data are consistent with what is known about the stimulatory effect of estradiol on oxytocin (Pedersen and Prange, 1979; Ochedalski et al., 2007) and suggest that elevating estradiol causes the release of pituitary stored oxytocin. This release is reflected in the urine as elevated oxytocin levels indicating that functional changes in oxytocin can be measured in urine. Juszczak and Stempniak (1997) found that female rats had lowered neurohypophyseal oxytocin levels and higher plasma oxytocin levels compared with basal levels 15 min after suckling offspring, suggesting that suckling caused the release of stored oxytocin from the neurohypophysis into the blood.

As with the measurement of circulating levels of oxytocin, determination in the urine requires validation of the methodology, the timing of release, and metabolic changes that may occur to the peptide excreted into the urine. For humans and the common marmoset, radiolabeled studies have indicated a time course from circulation into the urine as 30–60 min for peak levels to appear (Amico et al., 1987; Seltzer and Ziegler, 2007). This indicates that when examining responses to an event, the desired collection time would be within this window and the window appears to be consistent across the primate order. In plasma samples, oxytocin has a proteolytic enzymatic breakdown that occurs rapidly and requires an inhibitor added to the sample. Urine is acidic and limits the breakdown of oxytocin compared with the blood. However, there are metabolic processes that occur to oxytocin before it is excreted in the urine. HPLC separation and validation with the assay antibody can assure that the assay is exclusively measuring the intact oxytocin and/or peptides that stem from the oxytocin metabolism in the sample (Seltzer and Ziegler, 2007). Validations to test for this have been performed for several primate species (cotton-top tamarins: Snowdon et al., 2010; marmoset: Seltzer and Ziegler, 2007; humans: Seltzer et al., 2010; chimpanzees: Ziegler et al., unpublished data).

## Outlook for measuring endogenous peripheral oxytocin

Clearly, a complex and nuanced pallet of oxytocin, its releasers and its effects are emerging, which to some extent depend on the age, sex, previous experience, early attachment, psychiatric or psychological condition of the individual as well as the current social or non-social context, the social motivation or perception of individuals to the task at hand. In this review, we have referred to the three predominant theoretical approaches for examining how oxytocin effects or is released by social context or behavior. The most precise but most invasive is to examine the oxytocinergic system centrally. A less invasive option for examining the effects of oxytocin on behavior or social context is to administer oxytocin into the peripheral system. The least invasive option, and one suited to examining which behaviors and social contexts trigger the release of oxytocin, is to measure peripheral endogenous oxytocin levels. As pointed out in the review, all have their strengths and weaknesses. Thus, it is likely that to really understand the influences involved in the oxytocinergic system, a greater synthesis of these theoretical approaches will be necessary in the future. This is likely to be the case for determining how peripheral oxytocin actually links to central oxytocin, for further examination of the positive feedback mechanism between oxytocin and behavior or stress (Russell et al., 2003; De Dreu, 2012; Yee et al. in Olff et al., 2013), and for examining interactions between oxytocin and other hormones in different social contexts (see Heinrichs et al., 2003; Szczepanska-Sadowska, 2008; Skuse and Gallagher, 2009; van Anders et al., 2011; Neumann and Landgraf, 2012). Several recent studies that have produced particularly insightful results have combined inhaled oxytocin with subsequent measures of endogenous oxytocin and other hormones, such as cortisol, within particular social contexts (for review see Olff et al., 2013).

Studies using exogenous oxytocin have highlighted how a possible effect of oxytocin is to change individuals' social motivations or perceptions of a current social context, such that individuals that inhale oxytocin compared with those that do not, become more affiliative, attentive, prosocial or empathetic to the same social stimuli (e.g., Bartz et al., 2011; Chang et al., 2012; Parr et al., 2013; Shamay-Tsoory et al., 2013). Interestingly, studies that include measures of endogenous peripheral oxytocin indicate that individuals' social expectations or perceptions of a current social context may be a critical factor in determining whether or not oxytocin release is triggered. More research is needed to tease apart how much the release of oxytocin is dependent on the physical properties of a social interaction, such as how gentle or intimate is a bout of affiliative touch, or rather, how much the release of oxytocin is dependent on individuals' social perception or expectation of the social interaction, such as how trusting or intimate they perceive the bout of affiliative touch to be.

We stress that one strength of measuring peripheral, endogenous oxytocin lies in its non-invasive quality. Given that oxytocin is sensitive to stressors and several aspects of social context as well as the perceptions or social motivations of human and non-human primates alike, non-invasive sampling allows repeated measures to be examined in relation to particular social interactions or individuals within the subjects' normal social and physical environment (Holt-Lunstad et al., 2008; Crockford et al., 2013; Wittig et al., 2014). Importantly, variation in oxytocin response can be examined within-subjects to determine the impact of the type of social interaction, whether affiliative or trusting, and the relationship with the interaction partner, whether stranger, bond partner, in-group or out-group member. Another strength of non-invasive sampling is that several hormones can be measured simultaneously allowing examination of the influence of oxytocin interactions with other hormones on motivational states. Finally, as subjects can be sampled repeatedly over hours, days, or months whilst engaging in the normal influences and pressures of their social life, it is possible to examine complex questions such as how oxytocin contributes to the establishment and maintenance of a relationship, the related influence of anxiety and stress on social bond formation and maintenance, and the influence of social bonds on cooperation.

### Implications for human research

Oxytocin administration can lead to short-term improvements in the social interactions of people with psychiatric or psychological disorders (Macdonald and Feifel, 2013). However, first indications exist that chronic exposure to exogenous oxytocin can lead to a dampening of this effect and to even counterproductive results (Bales et al., 2013; but see Young, 2013). The results from Bales et al. (2013) to some extent reflect those of a study on women examining endogenous oxytocin levels, where high basal plasma oxytocin levels were associated both with relationship anxiety and attenuated plasma oxytocin levels following massage or positive emotion (Turner et al., 1999).

If oxytocin administration might not provide long term benefits, a more productive approach may be to invest in the identification of behaviors that trigger endogenous oxytocin release. Indeed, several recent studies show that changes in behavior achieved by oxytocin administration can also trigger endogenous oxytocin release and subsequent predicted changes in behavior. For example, in ewes, central administration of oxytocin induces maternal behavior (Kendrick et al., 1986) and vaginocervical stimulation induces lamb adoption (Keverne et al., 1983; humans: Seltzer et al., 2010). In rodents, central oxytocin induces grooming (Argiolas and Gessa, 1991) and in humans, under certain social circumstances, affiliative touch increases plasma oxytocin, as can vocal reassurance (Seltzer et al., 2010). In rodents, the primary form of food-sharing, lactation, is precipitated by central oxytocin (Ross and Young, 2009) and oxytocin is released during suckling across mammals (Argiolas and Gessa, 1991). In chimpanzees, food sharing between adults is associated with high urinary oxytocin levels (Wittig et al., 2014). Taken together, such studies suggest that oxytocin-associated social situations, such as food sharing or massage with bond partners, have the potential to function as natural oxytocin releasers. (Macdonald and Feifel, 2013) suggest that is likely to be more efficient to replace or augment some oxytocin administration therapy with non-drug therapies, especially in the long-term, for example, with cognitive therapy (Chou et al., 2012) or behavioral therapy. We suggest that including social interactions known to lead to endogenous oxytocin release in non-drug therapies may enhance therapy success.

It should be noted that research also begins to show that even when engaged in oxytocin-associated social interactions, like massage, endogenous oxytocin levels can be moderated by additional factors, such as the quality of the relationship between the subject and the interaction partner, the subjects' psychological state or perception of the current social context. More research is needed to determine key social contexts in which oxytocin administration is likely to be beneficial. Additionally, measures of endogenous peripheral oxytocin may act as a biomarker for the dynamic processes of social relationships, the strength, and valence of relationships and how these change over time.

## Author contributions

Catherine Crockford, Roman M. Wittig, Tobias Deschner, and Toni E. Ziegler all contributed to the writing of the manuscript.

### Conflict of interest statement

The authors declare that the research was conducted in the absence of any commercial or financial relationships that could be construed as a potential conflict of interest.
